# Entropy in Dynamic Systems

**DOI:** 10.3390/e21090896

**Published:** 2019-09-16

**Authors:** Jan Awrejcewicz, José A. Tenreiro Machado

**Affiliations:** 1 Department of Automation, Biomechanics and Mechatronics, Lodz University of Technology, 1/15 Stefanowski St., 90-924 Lodz, Poland; 2 Department of Electrical Engineering, Institute of Engineering, Polytechnic Institute of Porto, 4249-015 Porto, Portugal

In order to measure and quantify the complex behavior of real-world systems, either novel mathematical approaches or modifications of classical ones are required to precisely predict, monitor and control complicated chaotic and stochastic processes. Though the term of entropy comes from Greek and emphasizes its analogy to energy, nowadays, it has wandered to different branches of pure and applied sciences and is understood in a rather rough way with emphasis put on the transition from regular to chaotic states, stochastic and deterministic disorder, uniform and non-uniform distribution or decay of diversity.

This Special Issue originates from the 14th International Conference “Dynamical Systems – Theory and Applications”, held December 11–14, 2017 in Łódź (Poland), and addresses the notion of entropy in a very broad sense. The presented manuscripts follow from different branches of mathematical/physical sciences, natural/social sciences and engineering-oriented sciences with emphasis put on the complexity of dynamical systems. Topics like timing chaos and spatiotemporal chaos, bifurcation, synchronization and anti-synchronization, stability, lumped mass and continuous mechanical systems modeling, novel non-linear phenomena, and resonances are discussed.

An Analysis of Deterministic Chaos as an Entropy Source for Random Number Generators by Kaya Demir and Salih Ergün [1] presents the results of a comparison between bounded chaos, unbounded chaos and Gaussian white noise as a source of entropy for a random number of generators yielded by an analytical study of the autocorrelation and the approximate entropy analysis of the resulting bit sequences [2].

Information Transfer Among the Components in Multi-Dimensional Complex Dynamical Systems by Yimin Yin and Xiaojun Duan [3] provides a rigorous formalism of information transfer within a multi-dimensional deterministic dynamic system established for both continuous flows and discrete mappings.

Fractional Form of a Chaotic Map without Fixed Points: Chaos, Entropy and Control by Adel Ouannas, Xiong Wang, Amina-Aicha Khennaoui, Samir Bendoukha, Viet-Thanh Pham and Fawaz E. Alsaadi [4] presents the results of the first dynamic investigation of a fractional order chaotic map corresponding to a recently developed standard map that exhibits chaotic behavior with no fixed point. The authors use the approximate entropy measure to quantify the level of chaos in the fractional map.

Tsallis Entropy of Product MV-Algebra Dynamical Systems by Dagmar Markechová and Beloslav Riečan [5] provides an example of the mathematical modelling of Tsallis of product MV-algebra dynamical entropy to provide the entropy measure that is invariant under isomorphism.

A Novel Image Encryption Scheme Based on Self-Synchronous Chaotic Stream Cipher and Wavelet Transform by Chunlei Fan and Qun Ding [6] presents a self-synchronous chaotic stream cipher that ensures the limited error propagation of image data in the secure transmission of image data. The cipher is designed with the purpose of resisting active attack.

The General Solution of Singular Fractional-Order Linear Time-Invariant Continuous Systems with Regular Pencils by Iqbal M. Batiha, Reyad El-Khazali, Ahmed AlSaedi and Shaher Momani [7] proposes the use of the Adomian decomposition method based on the Caputo’s definition of the fractional-order derivative to obtain a general solution of singular fractional-order linear-time invariant continuous systems.

Quantifying Chaos by Various Computational Methods. Part 1: Simple Systems by Jan Awrejcewicz, Anton V. Krysko, Nikolay P. Erofeev, Vitalyj Dobriyan, Marina A. Barulina and Vadim A. Krysko [8] proposes an algorithm of calculation of the spectrum of Lyapunov exponents based on a trained neural network that can be used to compute a spectrum of Lyapunov exponents, and then to detect a transition of the system regular dynamics into chaos, hyperchaos, and others.

Quantifying Chaos by Various Computational Methods. Part 2: Vibrations of the Bernoulli–Euler Beam Subjected to Periodic and Colored Noise by Jan Awrejcewicz, Anton V. Krysko, Nikolay P. Erofeev, Vitalyi Dobriyan, Marina A. Barulina and Vadim A. Krysko [9] presents a theory of non-linear dynamics of flexible Euler–Bernoulli beams under transverse harmonic load and colored noise that has been extended to investigate a novel transition type exhibited by non-equilibrium systems embedded in a stochastic fluctuated medium. 

On Points Focusing Entropy by Ewa Korczak-Kubiak, Anna Loranty and Ryszard J. Pawlak [10] introduces the notion of a (asymptotical) focal entropy point allowing study of the local aspects of the entropy of non-autonomous dynamical systems.

Last but not least, Analytical Solutions for Multi-Time Scale Fractional Stochastic Differential Equations Driven by Fractional Brownian Motion and Their Applications by Xiao-Li Ding and Juan J. Nieto [11] presents a method of investigation of the analytical solutions of multi-time scale fractional stochastic differential equations driven by fractional Brownian motions that is continued in [12].
